# Inherited Platelet Function Disorder From Novel Mutations in RAS Guanyl-Releasing Protein-2 Confirmed by Sanger Sequencing

**DOI:** 10.7759/cureus.11708

**Published:** 2020-11-25

**Authors:** Abdulqader Al-Hebshi

**Affiliations:** 1 Pediatric Hematology Oncology, Prince Mohammed Bin Abdulaziz Hospital, Medina, SAU; 2 Pediatric Hematology Oncology, Ministry of National Guard Health Affairs, Medina, SAU; 3 Pediatric Hematology Oncology, King Abdullah International Medical Research Center, Riyadh, SAU; 4 Pediatric Hematology Oncology, King Saud Bin Abdulaziz University for Health Sciences, Riyadh, SAU

**Keywords:** inherited platelet disorder, rasgrp2, platelet dysfunction

## Abstract

Inherited platelet disorders (IPDs) are genetically heterogeneous rare disorders due to quantitative and/or qualitative abnormalities of the platelet. IPDs are often predisposed to significant medical complications. RAS guanyl-releasing protein-2 (RASGRP2) was recently identified as a gene affected in patients with platelet function defects and a bleeding complication. RASGRP2 codes for the protein CalDAG-GEFI RAS (guanyl-releasing protein-2), a guanine nucleotide exchange factor for small guanosine triphosphate(GTP)ase Rap1. We used Sanger sequencing to identify a novel function-disrupting homozygous mutation in RASGRP2 responsible for bleeding diathesis and platelet dysfunction in a patient.

## Introduction

Genetic defects of platelets constitute rare diseases [1], which affect the platelet number and function and often cause severe bleeding and other significant medical complications encountered in clinical practice [2]. The presentation may be variable platelet counts and bleeding episodes, complicating the diagnosis [3]. Identification of the underlying genetic defects in these disorders is complicated by the differences in the clinical expression of the bleeding symptoms in affected individuals [4]. ِAlso, it is problematic to establish the diagnosis based solely on function studies because, due to limited stability, immediate testing on fresh patient platelets is required. It is essential to use more advanced genetic testing such as next-generation sequencing, to be able to make an accurate diagnosis based on genetic information [5-6].

Glanzmann’s thrombasthenia is one of the most severe inherited platelet function disorders caused by recessive mutations in either integrin alpha-IIb (ITGA2B) or integrin beta chain beta 3 (ITGB3) leading to defects in the αIIbβ3 integrin and consequently to severely impaired platelet aggregation. RAS guanyl-releasing protein-2 (RASGRP2) was recently identified as a gene affected in patients with a platelet function defect and a bleeding complication [7-8]. It is strongly expressed in platelets and neutrophils, where its encoded protein CalDAG-GEFI facilitates the activation of Rap1 and subsequent activation of integrins [9-10]. A pathogenic variant in RASGRP2 has been associated with platelet-type bleeding disorder type 18 (OMIM: 615888). It is an autosomal recessive, nonsyndromic platelet function disorder that is associated with moderate or severe bleeding, similar to other disorders of the αIIbβ3 integrin [11].

To the extent of my knowledge this is the first case in Saudi Arabia that case reports the novel pathogenic RASGRP2 mutation c956C>T. (Pro319Leu) which is considered one of the causative variants for this rare autosomal recessive platelet-type bleeding disorder type 18 (BDPLT18).

## Case presentation

**Figure 1:**
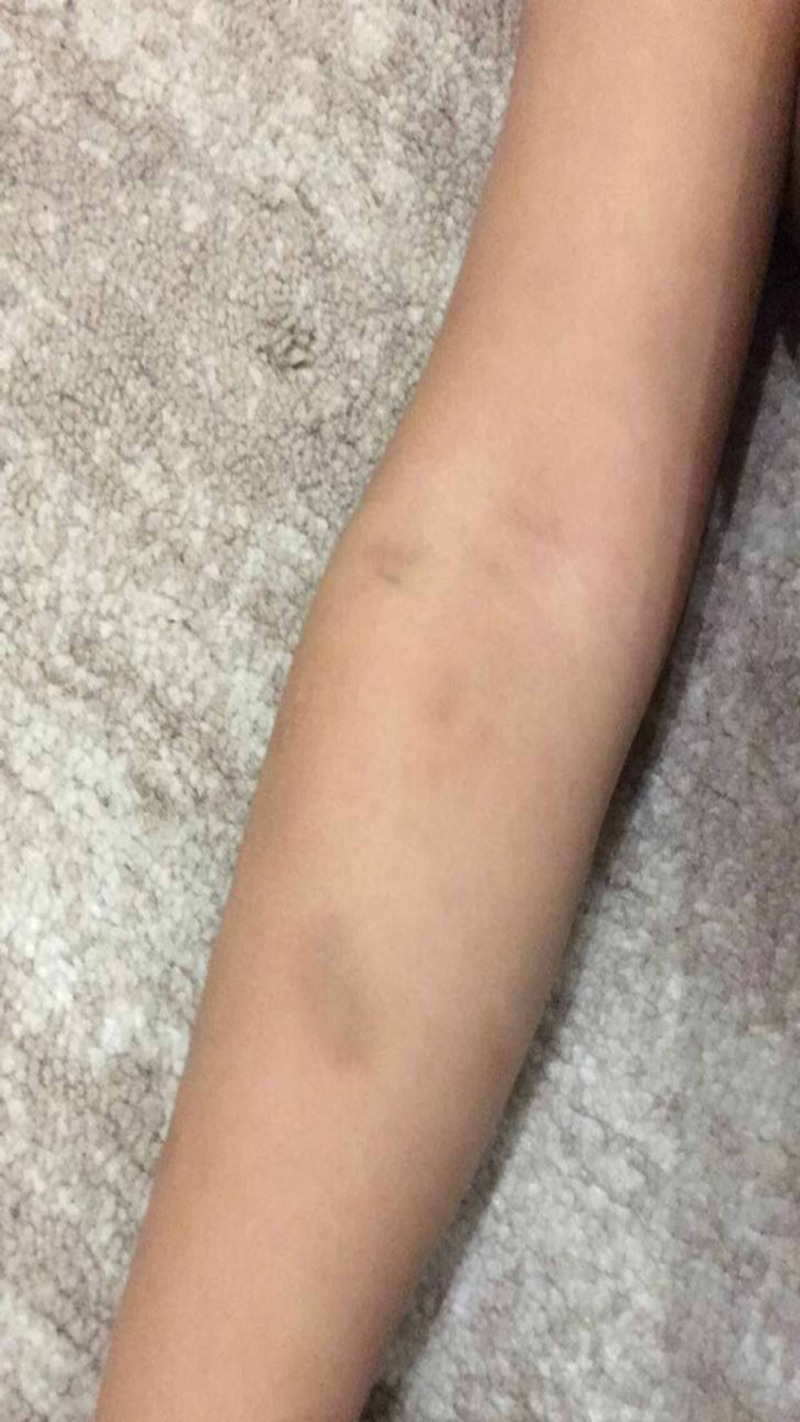
Shows multiple ecchymosis over the upper extremity

**Figure 2:**
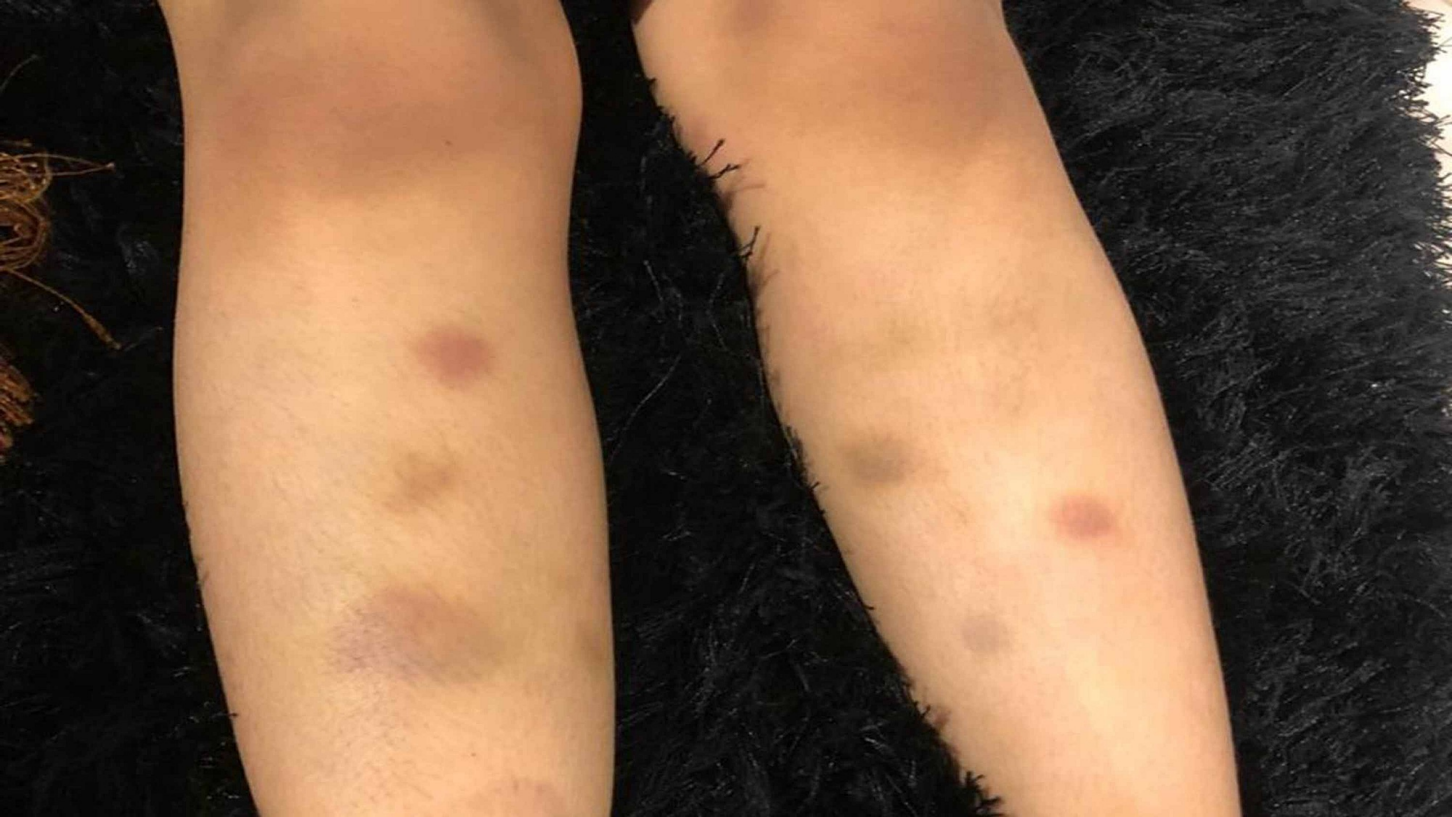
Shows multiple ecchymosis over the lower extremity

A three-year-old Saudi girl, with no known chronic illness, presented on the 2nd of October 2017 at the Emergency Room at Prince Mohammed Bin Abdulaziz Hospital, Ministry of National Guard Health Affairs, Medina, Saudi Arabia. She was accompanied by her parents, with a bilateral profuse non-stopping epistaxis lasting for a few hours. There was no bleeding from other orifices, she was conscious, alert, oriented, and calm, with normal vital signs (temperature 36.6^0^ C, respiratory rate 24/minute, oxygen saturation 96% on room air, heart rate 110 beats/minute, and blood pressure 121/77 mmHg), not dysmorphic, and was not cyanosed or distressed. She was bleeding actively (epistaxis) and there were multiple ecchymoses over her upper and lower extremities (Figure 1, 2) and abdomen with different colors and sizes. There was no hepatosplenomegaly or lymphadenopathy. Other systemic examinations were unremarkable. There was no history of trauma or drug ingestion. Another systemic review was unremarkable. After vomiting a large amount of digested blood, becoming paler and symptomatic with increased tachycardia, her hemoglobin started to drop from 11.5 g/dl to 8 g/dl (other complete blood count (CBC) indices remained normal), so she was transfused with packed red blood cells, fresh frozen plasma, as well as recombinant Factor VII, which were administrated to treat the symptomatic anemia and to stop the severe uncontrolled epistaxis. The Ear Nose and Throat team were consulted and they found multiple bleeding vessels, which were cauterized locally. Almost all bleeding completely stopped six hours post-admission.

The patient is a product of a full-term pregnancy, with a spontaneous vaginal delivery, no neonatal intensive care unit (NICU) admission, not known to have any chronic illness, not on any medication, and never received a blood transfusion before. There was a history of prolonged wound healing and profuse bleeding due to minimal injuries at the age of six months. Her parents are cousins and she has two siblings, all are healthy, with no family history of bleeding disorders.

Glanzmann thrombasthenia (GT) had initially been suspected, as this type of platelet dysfunction is common in our region. An initial bleeding-related work-up was done including CBC. The coagulation profile showed a normal platelet count, normal partial thromboplastin time (PTT), international normalized ratio (INR), normal Factor XIII, and the von Willebrand factor (VWF) panel was normal (Table 1, 2). Flow cytometry was done to rule out GT, which was normal. ITGA2B, ITGB3, and VWF gene sequencing were done and no relevant variant was detected. A platelet aggregation study (more than one reading) revealed a normal response to ristocetin and arachidonic acid but had an abnormal response to collagen, adenosine diphosphate (ADP), and epinephrine (Table 3). The peripheral blood smear was normal.

**Table 1:** Complete blood count (CBC). WBC, white blood cell; RBC, red blood cell; Hb, hemoglobin; Hct, hematocrit.

Test	Result	Reference range
WBC	13.9x 10^9^/L	(5-12 × 10^9^ cells/L)
RBC	4.25 x10^12 ^/L	(3.90-5.60x10^12^ /L )
Hgb	11.5 g/dl	(12 to 16 g/dL)
Hct	0.360 L/L	(0.310-.450 L/L)
Platelet	469 x10^9 ^/L	(150 – 450 × 10^9^ cells/L)

**Table 2:** Platelets aggregation test.

Test	Result	Reference range
Epinephrine aggregation	5 %	(70-90%)
Arachidonic Acid	81%	(70-90%)
Collagen aggregation	3 %	(70-90%)
Adenosine Deaminase	9%	(70-90%)
Ristocetin	84 %	(70-90%)

**Table 3:** Coagulation profile. VWF Ag, von Willebrand factor antigen; PTT, partial thromboplastin time; PT, prothrombin time; INR, international normalized ratio

Test	Result	Reference range
VWF Ag	65 %	(50-150 %)
VW Ristocetin Cofactor	59 %	(50-200 %)
Factor VIII	97 u/ml	(50-150 %)
PTT	32 sec	(26-41 seconds)
PT	12 sec	(11.0-14.5 sec)
INR	1 %	(0.8-1.2 %)
Factor XIII	83 u/ml	(70-140 %)
Thrombin time	18.3 sec	(14-19 sec)
Fibrinogen	2.5 g/L	(2-4 g/L)

The results of a genetic study (performed at Bioscientia Human Genetics, Ingelheim, Germany) identified a homozygous RASGRP2 VUS c.956 C>T p. (pro 319leu) which confirmed the diagnosis of BDPLT18, and based on this final genetic result, we recommended checking all the family members for the same gene. We found that all the other family members are heterozygous carriers of the same familial RASGRP2 VUS.

## Discussion

The biallelic pathogenic variant in RASGRP2 has been associated with platelet-type bleeding disorder type 18. It is an autosomal recessive, nonsyndromic platelet function disorder that is associated with moderate or severe bleeding, similar to other disorders of the αIIbβ3 integrin. There is a consistent laboratory phenotype of reduced aggregation response to adenosine 5′-diphosphate (ADP) and epinephrine, but no defect in dense granule secretion, distinguishing this disorder from δ-storage disease [11].

Pathogenic RASGRP2 variants are causative for autosomal recessive platelet-type bleeding disorder type 18 (BDPLT18) gene (605577) on chromosome 11q13. It is a rare hematological disease due to a CalDAG-GEFI deficiency (OMIM®615888). With a prevalence of <1/1000000 globally, the age of onset and the clinical course are observed in the infancy period. According to our literature review, Canault et al. reported three siblings, born of consanguine parents, with a bleeding disorder due to defective platelet function [1]. The patient developed mucocutaneous bleeding around 18 months of age. Features included epistaxis, hematomas, bleeding after tooth extraction, and menorrhagia. Bleeding times were increasing, and the patient’s platelets indicated a reduced aggregation in response to ADP or epinephrine. Canault et al. identified a homozygous mutation in the RASGRP2 gene (G248W; 605577.0001). The mutation was found by exome sequencing and segregated with the disorder in the family [12].

With the current case, we report a novel pathogenic RASGRP2 mutation c956C>T. (Pro319Leu) which causes an amino acid change from Pro to Leu at position 319. During the first presentation of our patient, Glanzmann thrombasthenia was initially suspected as the mother has a history of oozing after a cut wound but the sequencing analysis of ITGA2B, ITGB3 as well as von Willebrand gene was normal [13]. Further work was done using Clinical Exome Sequencing, which revealed a potential positive result for a variant of uncertain significance, which has been confirmed by Sanger sequencing as a homozygous variant of uncertain significance with the parents being carriers of the same variant. Consequently, the diagnosis of an autosomal recessive platelet-type bleeding 18 with the novel mutation was confirmed [14]. This type of variant was classified as a variant of uncertain significance (class 3) according to the recommendations of Centogene and ACMG (American College of Medical Genetics) (Table 4) [15].

**Table 4:** Centogene Variant classification (Based on American College of Medical Genetics recommendations).

Class1	Pathogenic
Class2	Likely pathogenic
Class3	Variant of uncertain significance (VUS)
Class 4	Likely benign
Class 5	Benign

## Conclusions

RAS guanyl-releasing protein-2 (RASGRP) variant c956C>T. (Pro319Leu) is uncommon cause of autosomal recessive platelet-type bleeding disorder type 18 (BDPLT18). This case was reported because of the rarity of such mutations affecting platelet dysfunction and aggregation causing hemostasis disorder.

I recommend performing RASGRP2 gene testing after excluding the common inherited platelet disorders (IPDs) like Glanzmann thrombasthenia because early detection of such rare causes will enable the physician to know the nature of the disease and how to anticipate the severity in comparison with other IPDs.
